# A Qualitative Exploration of Existing Reflective Practices Used by Undergraduate Dental Students in Paediatric Dentistry

**DOI:** 10.3390/dj10010001

**Published:** 2021-12-23

**Authors:** Faith Campbell, Kirsten Jack, Helen Rogers

**Affiliations:** 1 Academic Unit of Oral Health, Dentistry and Society, School of Clinical Dentistry, University of Sheffield, Sheffield S10 2TA, UK; faith.campbell@sheffield.ac.uk; 2 Faculty of Health and Education, Manchester Metropolitan University, Manchester M15 6GX, UK; k.jack@mmu.ac.uk; 3 School of Dental Sciences, Newcastle University, Newcastle upon Tyne NE2 4AZ, UK

**Keywords:** education, dental, graduate, education, dental continuing, teacher training

## Abstract

**Background:** Reflection is increasingly significant for dental students and professionals and is a continuing requirement of dental regulatory bodies. There is a paucity of evidence regarding how best to facilitate deep reflection for dental students. This study explored whether the use of clinical logbooks in undergraduate clinical attachments in Paediatric Dentistry was facilitating deep reflection. **Methods:** This qualitative study used individual interviews for data collection. This was conducted at the University of Sheffield with third year undergraduate dental students and clinical teaching staff. Interviews were immediately transcribed verbatim. A reflexive approach to thematic analysis was used to co-constitute the data, enabling the development of the thematic framework. **Results:** The sample compromised 10 students and 4 educators. Thematic analysis generated 4 key themes: understanding of reflection, preparation for reflection, importance of learning through experience, and suggestions for development. The findings indicated that students perceived that they were not being supported in engaging in deep reflection by the use of a clinical logbook and that greater preparation for reflection would be beneficial. **Conclusions:** The current study revealed that using clinical logbooks during clinical attachments in Paediatric Dentistry was not facilitating deep reflection. Further research is required to explore how deep reflection can be facilitated for undergraduate dental students undertaking clinical learning.

## 1. Introduction

Reflective thought may be defined as ‘the active, persistent and careful consideration of any belief, or supposed form of knowledge in the light of grounds that support it and the further conclusion to which it tends’ [1]. It enables one to direct their actions with foresight and to know what we are about when we act [1]. Reflection enables healthcare professionals to think critically about practice, supporting the development of their professional identity and enabling identification of learning needs. It can support self-awareness development, which enables self-monitoring and regulation within the healthcare culture [2]. Reflection can be undertaken in action, which occurs during experience where one can respond by modifying behaviour immediately and reflection on action after the experience, with consideration of the event with thought and feeling on this [3]. 

Reflective practice can help learners to bridge the gap between theory and practice, allowing them to find answers that they are unable to access through formal learning whilst exploring their emotions associated with their learning experience [3]. This is pertinent in workplace-based learning where learners may feel anxious or insecure regarding their contribution to patient care and their role within the healthcare team [4]. This may in turn present as a barrier to learning. Deeper and more meaningful reflection has been associated with improved self-awareness, supporting holistic and lifelong learning [5]. This is in contrast to superficial reflection, which is descriptive and less critical. Critical reflection demonstrates an awareness that actions and events are not only located within and explicable by multiple perspectives but are located in and influenced by multiple historical and socio-political contexts [6].

Deep reflection involves purposeful critical analysis of knowledge and experience in order to achieve deeper meaning and understanding [2]. These deeper levels of reflection are more difficult to reach and less frequently demonstrated [2]. It is helpful to engage in this deeper state of reflection because it has been proposed as the key to moving between surface and deep approaches to learning, and a deep approach to learning and reflection appears to be both fundamentally related and mutually beneficial [2].

Deep reflective thought can also engage the emotions [7]. This is important because positive emotions enhance the learning process and can help a learner to persist in challenging situations and provide a basis for new learning, whilst negative emotions can lead to irrationality, denial of future learning opportunities, and a failure to extract the learning that a given situation provides [7]. Negative emotions may also lead to increased dropout rates and negatively affect the students’ physical and psychological wellbeing [8]. Specifically in healthcare courses, the anxiety induced by a negative experience in a clinical placement can lead to increased attrition from the course [9]. These negative emotions may continue to be experienced when a learner is placed in the same situation. Through reflection, learners can acknowledge negative emotions, reducing the likelihood that they will disrupt future learning experiences [7]. This is important in Paediatric Dentistry, a speciality which can invoke additional anxiety in comparison to other dental attachment experiences [10]. 

Many international regulators of dental professionals require practitioners to demonstrate maintenance of their practice and knowledge according to current best practice as part of their standards for practitioners [11,12,13]. It is globally acknowledged that the development and maintenance of professional standards and skills involves rigorous self-assessment and reflection on one’s current practice [14,15]. 

Within the United Kingdom (UK), this idea has been developed further with greater incorporation of reflective practice within regulatory standards. Upon registration with the General Dental Council (GDC), registrants are required to undertake meaningful experiential learning on an ongoing basis, and should be able to explain the importance of critical reflection [16]. Furthermore, the GDC encourages dentists to be reflective practitioners, whereby they should consider their experiences to gain insight into their practice to support the continual improvement of the quality of their care. In 2018, the GDC introduced the Enhanced Continuing Professional Development Scheme (eCPD), a new cycle that changed the way that dental practitioners must undertake their continuing professional development (CPD) [17]. A key component of this cycle is reflection, with all practitioners required to keep an eCPD record including mandatory demonstration of reflection [17]. Outside of the UK, providers of dental education must support students to improve their performance by providing regular feedback and encouragement of reflecting on their practice [18,19].

Reflective practice is essential for undergraduate students throughout their dental education, with this being included in regulatory policy documents [18,20]. At the School of Clinical Dentistry (SCD), the University of Sheffield, UK, students must complete a formal written self-reflection in their personal logbook for every patient contact during clinical attachments in Paediatric Dentistry. 

Anecdotally, reflections in the logbooks are rarely used by students, and hence it has been questioned whether this is the most effective method in facilitating. There is limited evidence regarding how to appropriately support students in their reflective practice; an area that the present study intended to address. 

This study aims to explore staff and student perspectives on reflective practice by answering the research question; are current methods for reflection effective in facilitating the learning and personal and professional growth of undergraduate students undertaking clinical attachments in Paediatric Dentistry. The study focused on paediatric dental clinics, as students have described Paediatric Dentistry as invoking additional anxiety, and is therefore an area where the development of reflective practices may be particularly beneficial [10].

## 2. Materials and Methods

### 2.1. Context and Participant Recruitment

Ethical approval for this study was granted by the Ethics Committee at the University of Sheffield (reference: 034416). 

A qualitative approach was used, through semi-structured interviews with students and staff, which was followed by thematic analysis. 

Staff and students were purposively sampled to ensure that they had experience of using the logbook for reflective practice as a learner or educator, during the third year Bachelor of Dental Surgery (3BDS) clinical attachments in Paediatric Dentistry during the academic year 2019–2020 at the University of Sheffield. At this stage in their course students had passed clinical assessments on phantom heads to perform basic procedures on children, they had also provided dental care for patients for a year, including assessments, restorative procedures, dentures, and extractions on adults, but notably had no contact with child patients. During this previous year of clinical contact with patients, students had used a logbook for their reflective practice after each patient contact, so they were familiar with the current process of reflection. Reflection is a key requirement of the undergraduate course throughout the five years; thus students had been engaging in reflective writing through reports and verbal reflection. 

Both staff and student participants were recruited as they would have different but important experiences of the current method of reflective practice. This ensured that important data would not be missed and that future research resulting from this study could use data from both groups to inform any changes to reflective practice. 

**Figure 1:**
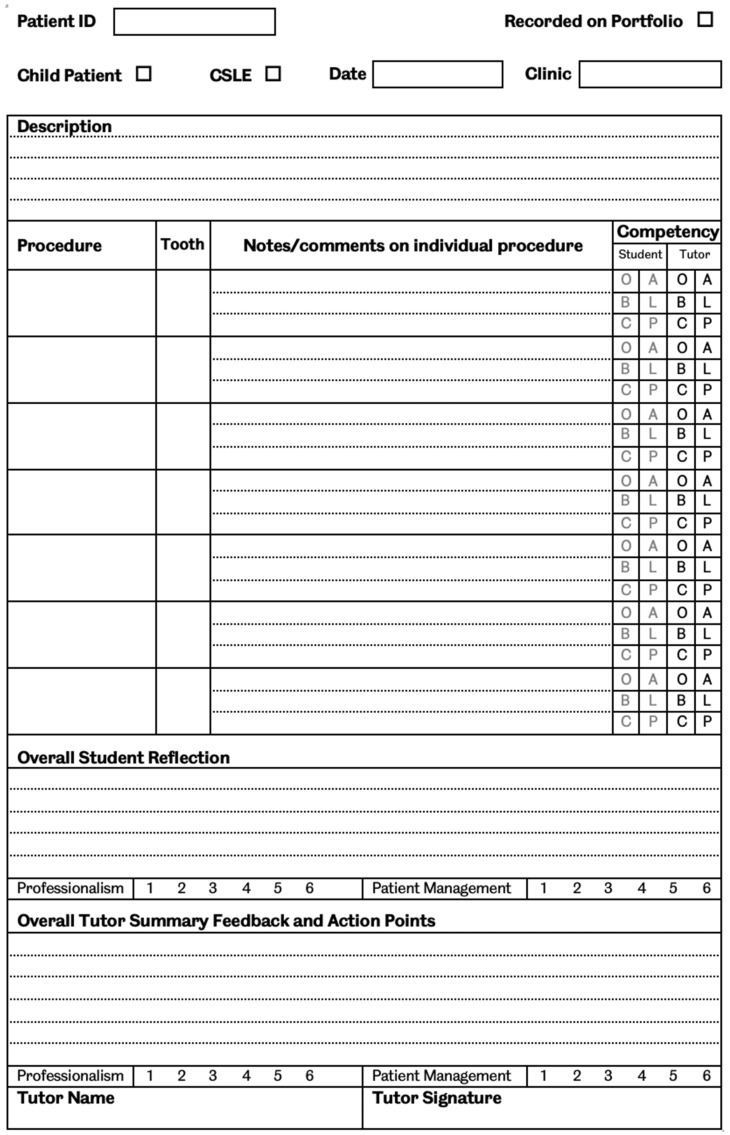
Sample page from the student logbook showing the unstructured space for reflection. CSLE—Clinical Skills Learning Environment, O—Observed, A—Assisted, B—Beginner, L—Learner, C—Competent, P—Proficient.

The logbook contains a blank space for an unstructured written reflection, which is completed on an open clinic at the end of a clinical session, during time which is not protected for this purpose and may overrun into personal time. This logbook is then handed in to SCD at the end of the rotation (Figure 1).

Students receive one lecture on the use of the clinical logbook and reflection prior to commencing clinical attachments. Clinical tutors who facilitate reflection receive no formal training on reflection or how to engage students in this using a logbook. These tutors are of varying backgrounds and include general dental practitioners, specialty trainees and consultants in paediatric dentistry. 

A General Data Protection Regulation (GDPR)-compliant, open-ended invitation to participate was sent by an administrative assistant to all eligible staff and students with no time limit, whilst further advertising was undertaken through social media. The participant information sheet was shared with the invitational email, and students were assured that their progress on the course would not be affected by their decision regarding participation. 

An online interview was arranged for those who responded to the invitation, following completion of a written consent form. Staff and students were advised that they would receive a £5 shopping voucher on completion of the interview, to thank them for their time.

### 2.2. Data Collection

**Figure 2:**
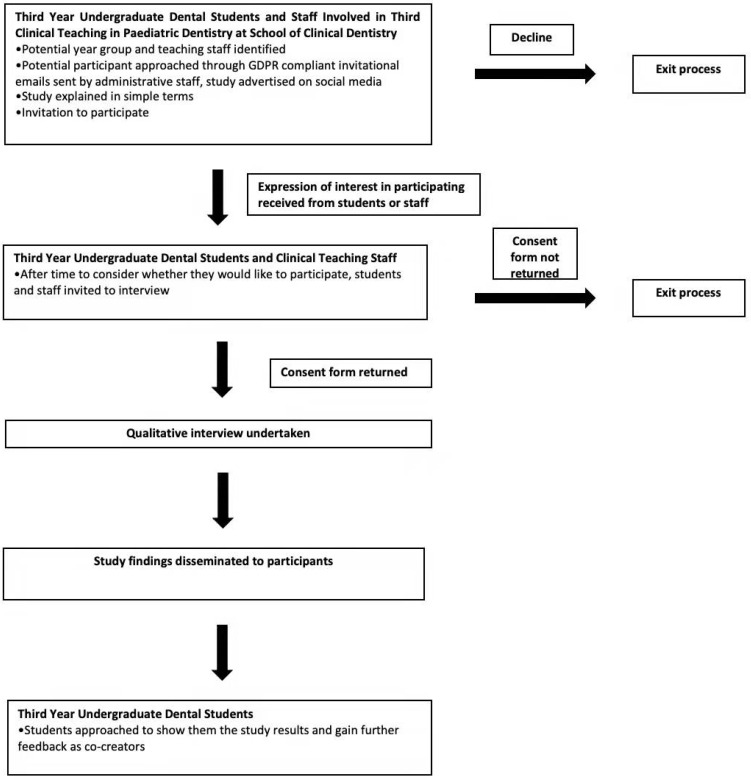
Participant recruitment flowchart.

All interviews were conducted through Google Meet (^©^Google, LLC, Mountain View, CA, USA) in a private room at the lead researchers’ home, in June and July 2020, due to the ongoing COVID-19 pandemic prohibiting physical interviews. The stages of student and staff recruitment are summarized in Figure 2. Interviews were chosen over focus groups to provide the opportunity to gain a deeper insight into individual participant perspectives on reflective practice, with group discussion not being necessary to illuminate the research topic. 

The study was explained to participants verbally prior to the interview, and consent was confirmed and recorded. Participants were advised that they could discontinue the interview or withdraw from the study at any time. Hermeneutic (interpretive) phenomenology was chosen as the approach for this research, as it enables understanding of the participants’ values, beliefs, feelings, and actions lived against the backdrop of their environment [21]. This ‘lifeworld’ approach contrasts with those which split participants from the world in which they live and is based on the philosophy of Heidegger [22]. Heidegger suggested that it is essential to understand the person’s lifeworld if we are to understand them as individuals [22]. This approach is in contrast to the descriptive phenomenological approach, which uncovers a ‘truth’ which is free from the researcher’s pre-suppositions [23]. Using the researcher’s interpretative lens enables understanding that might not be reached by bracketing off our pre-suppositions. This process is grounded in Heidegger’s description of the hermeneutic circle [22]. We start with our initial ‘whole’ of understanding, which is then exposed to the ‘parts’ of the student data. A different ‘whole’ is then reached and based on what we know already about the topic, combined with the student data. This circular process enables not only a description of student thoughts but also attempts to uncover the meaning behind them [24]. This is important in this study, for example, when considering the multiple contextual influences on students’ lives and how they reflect on their experiences. This approach has been used with success when researching reflective practices in other disciplines, for example, Nursing and Dental Technology [25,26]. 

Topic guides were developed to explore staff and student perceptions of the current method of reflecting within the paediatric dental clinic. The topic guides were piloted during the first interviews where both members of the interviewing team were present, and adjusted as necessary to produce a more logical structure, and minimise repetition. The topic guide was subsequently developed iteratively after each interview to prompt exploration of new themes.

Any students who had been taught by the interviewer involved with 3BDS teaching (FC) were interviewed by the alternative interviewer (HJR). Interviewers were both female, and at the time of study one was a post CCST specialist and honorary clinical lecturer in Paediatric Dentistry and the other was a clinical teacher in Paediatric Dentistry, with participants being aware that they were both staff members, the interviewers had a working relationship with staff members but no relationship with any student participants. The former has experience and formal training in qualitative interviewing, whilst the latter did not. The participants were aware that the intention of the interviews was to gain insight into their views on reflection. Interviewers wore non-clinical attire for the interviews. Both interviewers had interest but no bias relating to the research topic. 

Consideration was given to potential power issues between the researchers conducting the interviews and participants, to minimise conflict of interest the researcher involved in teaching at 3BDS level did not conduct any interviews with any students whom they had taught. The researchers were and are not involved in student progress committees or any senior level of student management. Staff participants were known to the researchers. 

A digital voice recorder and the recording function in Google Meet were both used to record the interviews, which were subsequently, transcribed verbatim. Field notes were made during every interview. Participants were recruited and interviews undertaken until no new ideas emerged and it was agreed that data saturation had been reached.

### 2.3. Data Analysis

Thematic analysis was conducted on the transcripts of the interviews, which were organised using NVivo version 12 (^©^QSR International PSY Ltd., Melbourne, Australia) software by one of the researchers (FC) following data familiarisation. This was undertaken immediately after each interview, alongside use of a saturation grid. Data were initially analysed independently by two researchers (FC and KJ). One researcher is a Professor of Nursing Education and is a National Teaching Fellow and an experienced qualitative researcher of the topic area. The lead researcher has practical experience of teaching and facilitating reflection with undergraduate dental students. Both researchers discussed their analysis to reach agreement on themes using a constant comparative approach until consensus on the meaning of the data was reached [27]. The final results of the analysis were then shared with experienced academics at The School of Clinical Dentistry at the University of Sheffield. We applied the standards for rigour as described by van Manen, which included the presentation of a clear audit trail and the use of verbatim quotes, which enabled the meaning and context of the participants’ experiences to be clearly described [28]. 

A reflexive approach to analysis enabled the combination of experiences of the research team with those of the participants, to co-constitute the data [22]. This enabled the development of the thematic framework. This approach required the team to move from the whole of the data to individual parts and then back again, to develop a different understanding of the phenomenon. Throughout the process, all explanations of the data were considered through discussion and the main elements of each theme were explored and described using original data excerpts. Using a ‘whole-parts-new whole’ approach enabled the team to discover new meanings about the phenomenon of dental student reflective practices, and how they are experienced by both students and educators. Key themes were identified and relayed back to the participants to ensure their statements have been interpreted as intended.

## 3. Results

There were ten student respondents, and all were interviewed. Any that had been taught by members of the research team (FC) were interviewed by HJR, who had no involvement in teaching 3BDS. Four staff members, excluding members of the research team who had taught 3BDS during clinical attachments in Paediatric Dentistry, responded, and all were interviewed. No repeat interviews were conducted. There were no non-participants and nobody from either group dropped out of the study. Data saturation was reached when all four suitable staff participants and ten student participants had been interviewed. The transcripts and associated themes were returned to the participants, and all felt that their experiences were accurately represented in each. Participant characteristics can be seen in Table 1.

Several of the participants from both staff and student groups had a basic understanding of reflective practice, in terms of exploration of what went ‘right or wrong’. Many students described the logbook as being unhelpful to support deep learning from experience. However, several participants were able to articulate the importance of reflective learning and offered suggestions about how this could be supported in the programme. 

Analysis of these qualitative data resulted in four overarching themes (Table 2):

1. Understanding of Reflection
2. Preparation for Reflection
3. Importance of Learning Through Experience
4. Suggestions for Development

### 3.1. Understanding of Reflection

The data revealed a varied understanding of reflective practice. Several participants described the concept in basic terms, for example, to think about what had gone well or badly in their clinical sessions. All participants described reflection as something that occurred after an experience. The following participants described reflection as a descriptive task and one which could serve as an aide memoire for future:

*I write something first about what I think has happened, and, bits that I think had gone well, bits that have gone badly or things, usually I like to pick out something which was…where it’s something new to me, and which I just learnt, so, because it’ll help me remember better, and then the tutor then looks at it, and then gives me feedback based on my reflections*

(Participant 2, student)

*I think that reflection is pretty much just us writing in our clinic books… I find it quite useful to look back sometimes … we don’t have ‘paeds’ very often so I look before my next rotation if I have the book … generally, I just try and write something I learned in the session to trigger my memory next time*

(Participant 3, student)

*Reflect on what we’ve done on clinic and reflect on how you feel like it’s gone with the patient … the treatment, what you actually did and if that was any good*

(Participant 5, student)

For the following participants, reflection was an opportunity to identify improvements which could be made to their practice and ensuring that positive aspects of practice could continue to be developed. For the educator, reflection enabled not only identification of positive practice, but also a consideration of the reason why something went well:

*So I suppose it’s looking at what you’ve done on the clinic, your actions and clinical work and sort of seeing how you could have, what you did well, what you didn’t do so well and sort of trying to act on what you didn’t do so well to make it better for next time, so it’s more about trying to improve future clinical practice*

(Participant 6, student)

*So, you can look back and look at everything that went well and that didn’t so go well, and how you go on from that … also, so not just improve on the stuff that went wrong but keep on doing things right*

(Participant 7, student)

*Just about how you…when you’ve done a job … and then it’s, why did it go well, what have I done different…so reflection to me is just checking yourself, looking back at what you’ve done, trying to put into place things that you can follow to make it better next time*

(Participant 3, staff)

For the following participants, reflection was viewed as a therapeutic process although this was determined by the actions of the educator during the joint discussion. Reflection enabled students to track growth in their confidence and this process was either supported by positive and constructive comments or reduced by the educators focussing only on negative aspects:

*Sometimes … you might beat yourself (up) or just putting yourself down and sometimes, reflection can be positive as well, almost like a mini counselling session*

(Participant 1, student)

*I think that to somehow track how your confidence grows … be able to get a better overall picture … because I know…everyone knows that we’re probably nervous. (Laughter) Yeah, I think it might be helpful as well to the tutors that may look at it afterwards and see that, read, ‘Oh, I was really worried about that’ and then they could either silence those worries and say, ‘Well, you know, you did okay,’ or, ‘Actually, well, if you do it like that next time,’ and you might not feel so worried or whatever*

(Participant 5, student)

*…it depends which tutor you have. Sometimes, they just focus on what you’ve done badly, and I think a lot of people, myself included, find that quite off-putting … you shouldn’t take it as a personal attack but it almost is because it’s nice to begin with a positive and have the bit that you need to improve on and then end with a positive as well because I think people engage with a bit more than if you’re just told, “Right, so you didn’t do this well,” and that’s it*

(Participant 6, staff)

### 3.2. Preparation for Reflection

None of the student participants could remember having any formal support to develop their reflective thinking, describing it as something they picked up from their peers. This left some participants unsure about the standard and depth of their required reflective thinking. Within the course students do not presently have formal preparation for reflection in the logbook, a lecture on logbooks is given but there is no specific teaching on the importance of reflection and how to do this:

*It’s just something you develop whilst you’re on clinic work, whether it be actually treating the patient or seeing my peers work on patients and then seeing how they discuss the performance with the tutor … it’s just sort of something you pick up. In terms of formal teaching, I don’t recall having marks on it*

(Participant 4, student)

*… we’re not taught how deep to go with it. So I think with myself I go quite, I try and be quite detailed with it because it’s only the more detail we put in, the more I can identify what I’ve done wrong but I think a lot of people don’t really take it very seriously or they don’t go that much in detail so some people I’d seen on clinics say I’ll be better at this next time but they haven’t said how they need to be better at it. And I think that a lot of people just go very superficially into it*

(Participant 6, student)

*I feel like I make it up as I go along. I thought like (laughter) you know, when you’re at school and it’s like two stars and a wish. I feel like (laughter) it’s a little bit ambiguous, and we just…I kind of just write anything down.*

(Participant 2, student)

The following participants described the logbook as a way to document observations rather than reflection on their practice and were unclear about how this method of reflection should be used. Further to their lecture on clinical logbooks, students are advised on how to use their logbook during their clinical induction to clinics on Paediatric Dentistry by their clinical tutor, therefore this is dependent on the educator understanding and explaining how to use the book for effective reflective practice. There was also uncertainty about the role of the educator and the type of feedback they were meant to provide to develop the students’ reflections, and educators are currently not formally prepared on the use of the logbook to reflect and develop these skills:

*A lot of the time … I’ve written, “observed child communication and assisted full assessment and learned about hypomineralised molars and the appearance of them”. I suppose that is an observation rather than a reflection. And I know that that’s something I’m guilty of doing in all of my logbooks…*

(Participant 6, student)

*I think there is confusion among tutors and the students about what exactly you’re meant to put in there, and what the tutors- what feedback the tutor’s meant to give you as well*

(Participant 2, student)

The reflective process occurred at the end of a clinic and led to a rushed, unmeaningful experience for the students. The timing meant that students were left with no time to prepare their reflective thinking to enable them to share it with the educator. This led to students finding the reflective experience stressful and needing more time to prepare for the conversation:

*…you feel like you have to get something down in order to leave the clinic, and so, when you haven’t had, you know, that maybe a session wasn’t as interesting or it’s something that you’ve already done before, the fact that you have to, you know, it’s compulsory to reflect in order to leave, you just think, ‘Oh, okay. I don’t really have anything to say…’ reflection for me, it’s more of a…I don’t know, maybe I’m being too philosophical, but it’s more of a personal thing. And so, yeah, sometimes, I don’t feel like reflecting*

(Participant 1, student)

*It depends on how rushed they all feel because they will leave it to the last, oh, I put it on the book, can you sign all these papers, and then it’s all stressful, …you don’t have time to talk to them, you don’t have time, you just- they write their reflection, you write yours, you’re there for like a 2-min chat, it’s not…that’s not ideal I don’t think, … so you just haven’t got time. It’d be nice to have plenty of time to do it*

(Participant 3, staff)

In contrast, the next participant felt that the logbook facilitated conversations with students, was useful for prompting learning points, and enabled immediate feedback on practice. The preceding clinical sessions were adequate preparation for the reflective conversation, although in this excerpt this is described as feedback rather than a joint reflective process:

*Sometimes things happen during the sessions that you maybe need to really brought up that … think about this, so I think there’s lines that they need—it’s important that if something important happens in that session that really needed to be given feedback on*

(Participant 2, staff)

For the following participants, there were concerns about the safety of reflecting at the end of the clinic session when their peers and other educators might be present. The timing and environment led some to conceal reflection on their feelings with concerns about confidentiality:

*… you got a queue of people waiting to get their books signed and waiting to speak to the tutor …and, you know, how you felt if you’re feeling a bit emotional or upset, you may not feel like it’s a safe environment to disclose those things … your peers who, you know, are behind, you don’t want them to know how you’re feeling or see that you’re upset or bothered by things*

(Participant 1, student)

*They probably do socially modify what they write if I’m standing over them. But they tend to just write it themselves, and they write very grey stuff. I don’t think they write enough for anybody to judge them on*

(Participant 1, staff)

Only one student participant disagreed about the need for a safer space to reflect:

*… because I know tutors, at the end of the day, they just want you to improve; they’re not going to criticise you for being negative or picking up things that you can improve on. So, I don’t really think it would make a difference (having a safer space)*

(Participant 4, student)

### 3.3. Importance of Learning through Experience

The following participants understood the importance of reflective practice, although tended to view it to make improvements to patient care, rather than an exploration of their personal thoughts and feelings:

*I think it helps you with being able to provide better care for your patients. So, as I said before, areas that you need to improve on can be highlighted through your reflection if you do it properly. And that’s only going to advantage all the patients and the people that you interact with. And, you know, it could highlight that you need to do more CPD or go on courses …*

(Participant 1, student)

*We have a standard professionally that we need to fulfil when treating all of our patients. So, once you have a standard in mind, that is really valuable to see where you measure up against it. Whether you’re just passing it or you need to work on something or everything’s going well, it’s always important to make sure that the patient gets that standard of care and you’re trying your best to provide it*

(Participant 4, student)

*Because we need to improve. I think you need to reflect, look at what actually you could have done better and what did go well? And so, you can sort of build up on that*

(Participant 5, student)

*I think a lot of people think that the review and assessment bits and reflection is all about just making yourself better but people I don’t think realise that it’s actual requirement of the GDC through professional development*

(Participant 6, student)

The learning depended to a great extent on the partnership between the students and the educators. Opportunities for deep reflection were lost if educators provided superficial feedback. Engaging in peer feedback was viewed as a helpful way to identify improvements which could be made to practice, in contrast to feedback from educators:

*…You could think that you’ve done an amazing job. But really, the tutor hasn’t really looked at it, you know, doesn’t have anything else to say other than, ‘Oh yeah, good.’ And so, the opportunity to reflect on a deeper level isn’t always there I’d say, because, you know, your knowledge on what’s good and what’s bad as a student isn’t always, you know, as good as it could be obviously*

(Participant 1, student)

*I’ll talk about how I think it is then, but then often the tutor will be able to point things out to me, and then I guess it’s a nice feedback they give me as well, it gives me a better understanding of what I’ve done, yeah*

(Participant 2, student)

*I think a lot of the time, people find it difficult to identify what they have done wrong in the session so they need to have some element of peer review. So maybe that partner would sit with them and talk to them honestly about what they think they didn’t do so well as well what I did well …*

(Participant 6, student)

### 3.4. Suggestions for Development

Several participants had suggestions about how preparation and support for reflective learning could be enhanced. For the following participants, a more formal structure would be beneficial to support deep reflection:

*For me, maybe just having maybe just a little bit of guidance or like a point on, okay, something, what went well, what didn’t go well. Just something like that, just something to hint you to think a little bit more deeply, and just to make it a bit more something that is sit down, and…yeah, just think about it a bit more instead of just a gap. To keep the gap as well just for any other random comments, but more of a guided reflection perhaps*

(Participant 1, student)

*I think a breakdown of the reflection box … what went well, what didn’t go well, what did you learn today that forces you to write something. But I like the idea of, well, potential idea of not having to do it every session, doing it so many times a session. Because then, it’s not like a forced, ‘Well, I’ve got to write something because,’ you know. So, I think that would take that away. And actually, then, you’d maybe have, I don’t know, like for a four-week rotation, have two really good things you’ve actually found really interesting…*

(Participant 5, student)

*I suppose I write in the reflections what I need to, what things I’ve learnt about and what things I need to go away and learn about myself and I suppose that greater awareness of what knowledge you’re lacking so that you can work on it. It does improve your work a bit more. But I think, the reflections, if we’re told how to properly reflect could be more valuable than they are at the moment*

(Participant 6, student)

Making reflective activity optional or having the opportunity to reflect with peers were suggestions from the following participants:

*Is it compulsory to reflect on every clinic? Maybe just making it, you know, an optional thing that I have a reflection. You know, the tutor can always give comments… Do you want to reflect on this procedure or…? Yeah, maybe making it an optional thing*

(Participant 1, student)

*One thing that would be more useful with the peers, is that peers see you all the time. Whereas the staff members, is looking after two to four units at a time. So, I think the peer review would be more valuable because they’ve seen you from when you’ve brought the patient in to when you’ve sent them out*

(Participant 6, student)

For the following participants, having the flexibility of an app or having an online option were suggestions to engage students further in reflective practices:

*So, I don’t know if you’ve heard of these apps. So, the applications where you just basically track what you eat during the day… on the e-portfolio, I can see the number of treatments, professionalism, patient management, in a similar way like in a kind of a chart representation. So, something like this in terms of the layout on the e-portfolio like, yeah, the professionalism, average management; if that could be translated into something that we could access really quickly like an app, that might be another option*

(Participant 1, student)

*As more and more things go online if these things can be done online and they can submit them there. And the tutors could have access to look at things and to help them because it means they can go away and do it in their own time. I don’t necessarily reflect straightaway because if something happens on clinic that I haven’t, if I haven’t had a great experience I’m a bit wound up at the time so, my thinking’s not particularly clear so I wouldn’t think about it until later when I’m a lot more calm and reasonable and I think that would be better for the students because they’ve got time and they can access something where they can look at the questions and start to think about it*

(Participant 4, staff)

## 4. Discussion

This study has explored student and staff perceptions on current methods used to facilitate reflective practice for undergraduate dental students undertaking clinical attachments in Paediatric Dentistry. This was done using qualitative semi-structured interviews and thematic analysis. The participants had a limited understanding of reflection, and their experiences suggest that the logbooks do not support deep reflection.

There are a number of strengths to this study; notably, the rigour of the study design, and the experience and varied skills of the research team. Furthermore, this novel study highlights the importance of reflective practice in supporting students to deal with stress and anxiety, which is likely to be further exacerbated by the COVID-19 pandemic. Whilst this study focused on the use of reflection in paediatric dental rotations, the findings may be relevant for other undergraduate rotations that use a similar reflective process.

The existing relationship between the interviewers and staff participants was an unavoidable limitation in this study, in that it may have precluded open discussion. Furthermore, as all members of the research team had prior and ongoing experience of undertaking reflection in a clinical setting as learners and educators, respectively, the questions asked and interpretation of responses may have been subject to bias. Purposive sampling of one year group within one discipline in one dental school restricted the diversity of potential participants. There was an imbalance in the gender of the participants, as 8 of the 10 students and all four staff participants were female, which is 12 out of a total of 14 participants. This imbalance should be acknowledged when considering the findings. 

It was evident that understanding of reflection was extremely varied, for some was elementary, focusing on improvement in clinical skills and knowledge, and performed by all on action as opposed to in action. Limited understanding appeared to be linked to a perceived lack of support for students in developing reflective skills. There is evidence that individuals with a more linear, superficial understanding of the reflective process failed to improve the quality of their reflection compared with those with a deep understanding, supporting the need for greater understanding of reflection within staff and student groups [2]. Moreover, it is acknowledged that reflection is a fundamental aspect of the learning cycle. Schon described the positive impact of teaching reflective practice to learners, to successfully equip them for their real-world practice [29]. As such, there is a need for reflective practice to be taught effectively to facilitate learners’ professional development. Superficial approaches to the teaching of reflection have been linked to superficial levels of reflective thinking in students [1]. Approaches to the teaching of reflection are often inconsistent and generally misunderstood. Furthermore, there is little guidance to support educators in understanding reflective ability in learners, with students being found to suffer from an overload of reflection particularly in the early years of their learning programmes [30,31]. 

Participants appreciated the clinical logbook for prompting immediate feedback, discussions, and learning, though not for the intended purpose of deep and purposeful reflection. The focus of the reflective exercise was, in general, perceived as being for ‘improvement’ rather than the articulation of the development of relational skills, which are very important in dental practice. This is important, as these skills are enriched through discussion and reflection, and by not being aware of the purpose and benefit of this deep reflection, students were unable to access the occasion to develop. The opportunity to communicate with the clinical tutor was seen as valuable by both staff and student participants. It is acknowledged that logbooks facilitate immediate and ongoing communication between learner and educator in the clinical environment, alongside providing a feedback loop for evaluating the learning activity and a method of continuous assessment [32]. However, logbooks can often be inadequate for reasons such as a learner perception of logbooks being boring, bureaucratic, and an exercise in collecting signatures with no consequence for improper completion and a misalignment of clinical experience and logbook requirements [33]. This is found in the present study, where reflecting in the logbook was perceived as almost bureaucratic due to its compulsory nature, with the logbook feeling like a tick box exercise. Considering this alongside the feeling that there is not always something to be reflected upon, thus a desire for optional reflection, any interventions to change reflective practice should be flexible in how and when students reflect to respond to this feeling of reflection being laborious.

Lack of privacy, tutor engagement, and time were described as further barriers to engaging in deeper reflection. These findings concur with current literature that suggests that the setting and time available for reflection affect the depth of reflection undertaken [3]. Reflection was always discussed as occurring at the end of a clinical session rather than during. This therefore suggests that there may be value in dedicating specific time within and during clinical teaching sessions for reflection to be undertaken so that it is given greater importance and not perceived as an afterthought, as it was described by many participants, allowing both reflection in and on action to occur. Reflection in action is also described as ‘thinking on our feet’ and this is important, as it allows one to connect their feelings with experience and build an understanding of the situation in which they find themselves [3]. Continuing this practice can help to improve the quality of patient care. It is recommended by the NHS staff and learners’ mental wellbeing commission that healthcare learners and professionals have protected time and access to safe spaces in which reflect on clinical experiences [9]. Some students felt that reflecting optionally and with peers if they wish would be beneficial, supported by literature stating that deeper reflective thinking can be fostered by mutual support of group members, the opportunity to consider experiences more deeply, and learn from the experience of others [2]. 

The study was undertaken in the paediatric dental clinic, where dental students are known to experience significant anxiety about performing their first paediatric procedures [10]. More broadly, dental students experience significant stress and anxiety throughout their studies, with a significant impact on their mental and physical wellbeing [34,35,36]. Moreover, students undertaking undergraduate healthcare courses often have increased stress due to the academic and clinical demands of their course and are less likely to report or seek health for mental health conditions, fearing the consequences of doing so [9]. Student anxiety can also be an indicator for poorer academic performance and may exacerbate patient anxiety during treatment [34,35]. During the current COVID-19 pandemic, dental students are experiencing heightened levels of anxiety and worry about their studies suffering [37]. Reflection was valued by staff and students and was described as providing a ‘mini-counselling session’ which helped with stress and worries about clinical encounters and performance. 

Emotions can have significant effects on students’ productivity and interpersonal perceptions, with negative emotions negatively affecting both the current and future learning experience and students’ wellbeing [8]. Greater subjective wellbeing, defined as the cognitive and affective evaluations of ones’ life, also correlates with higher academic performance in further education [38]. The regulation of emotions is also highlighted as a key skill for successful healthcare professionals in effectively delivering high quality care [9]. Thus, it is reasonable to assume that the exploration of emotions through deep reflection is a vital tool in fostering a healthy and effective learning environment. 

With so many student participants already undertaking reflection in their personal lives, it became evident that participants recognise the importance of deep reflection. Suggestions for improvement included making a safer space for reflection and providing more formal guidance on reflection relating to the need for more formal support in developing knowledge and understanding of reflective skills. In this post-Bawa-Gaba era, following a key case whereby a doctor in training had their reflective records evidenced against them in court, healthcare professionals are finding written reflection to no longer be a safe space to reflect, and have called for the opportunity to use different methods [39].

Although the findings of this study may not be generalisable to entire student populations in all institutions, the most important aspect of the research was capturing the hermeneutic stories of the participants and thus the data speak for themselves. The findings of this study are valuable as they can now be used to justify and guide an intervention to effectively facilitate deep reflection for undergraduate dental students. Rolfe refers to ‘wicked’ problems, which are those complex challenges faced daily by healthcare practitioners which cannot simply be solved by simply applying best evidence to the situation, and cannot be fully understood until they attempt to solve it [40]. These situations are not amenable to simple application of past experience or current evidence, and reflective practice, specifically experimenting in action, is the best hope of successfully managing them [40]. Schön argued that when the practitioner reflects in action, they become their own researcher [3]. In an ever-demanding healthcare sector, where no amount of theory or knowledge of evidence will enable one to manage these problems, it is vital to create a generation of practitioners who are their own theorists and researchers by generating and testing hypotheses on the spot in the form of practice interventions [40].

## 5. Conclusions

The results of this study show that in its current form, the logbook is not facilitating students in engaging in deep reflection, with staff and students feeling that greater understanding of and support in reflection would be beneficial in improving clinical experience and learning. 

## Figures and Tables

**Table 1:** Participant and Interview Characteristics.

	Student Participants	Staff Participants
**Experience**	All 3 BDS	Clinical Tutor in Paediatric Dentistry Specialty Trainee and Clinical Tutor in Paediatric Dentistry Clinical Tutor and Specialist in Paediatric Dentistry Professor and Consultant Paediatric Dentistry
**Number of Participants**	10	4
**Gender (F/M)**	8/2	4/0
**Mean and Range Interview Length (Mins)**	25:87 (17:59–33:23)	12:10 (8:57–16:20)

**Table 2:** Summary of themes.

Understanding of Reflection	Preparation for Reflection	Importance of Learning through Experience	Suggestions for Development
Descriptive learning task Identifies improvements Therapeutic process	Informal process Uncertainty about logbook Inadequate time Safety aspects	Improvement to patient care Educator/Student relationship Peer feedback	Formal preparation Optional activity Online options

## Data Availability

Anonymized data obtained through this study are available upon reasonable request to the corresponding author. Data are not publicly available for privacy reasons.
